# Origin of the vasculature supporting growth of primary patient tumor xenografts

**DOI:** 10.1186/1479-5876-11-110

**Published:** 2013-05-03

**Authors:** Bonnie L Hylander, Natalie Punt, Haikuo Tang, Joanna Hillman, Mary Vaughan, Wiam Bshara, Rose Pitoniak, Elizabeth A Repasky

**Affiliations:** 1 Department of Immunology, Roswell Park Cancer Institute, Elm and Carlton Streets, Buffalo, NY, 14263, USA; 2 Department of Molecular and Cellular Biology, Roswell Park Cancer Institute, Elm and Carlton Streets, Buffalo, NY, 14263, USA; 3 Department of Pathology, Roswell Park Cancer Institute, Elm and Carlton Streets, Buffalo, NY, 14263, USA; 4 Current address: Department of Oral and Maxillofacial Surgery, the Affiliated Stomatology Hospital of Sun Yat-sen University, Guangzhou, Guangdong, 510055, China

**Keywords:** Patient tumor xenograft, Vascularization, Primary xenograft

## Abstract

**Background:**

Studies of primary patient tumor xenografts grown in immunodeficient mice have shown that these tumors histologically and genetically closely resemble the original tumors. These patient xenograft models are becoming widely used for therapeutic efficacy studies. Because many therapies are directed at tumor stromal components and because the tumor microenvironment also is known to influence the response of a tumor to therapy, it is important to understand the nature of the stroma and, in particular, the vascular supply of patient xenografts.

**Methods:**

Patient tumor xenografts were established by implanting undisrupted pieces of patient tumors in SCID mice. For this study, formalin fixed, paraffin embedded specimens from several types of solid tumors were selected and, using species-specific antibodies which react with formalin fixed antigens, we analyzed the species origin of the stroma and blood vessels that supported tumor growth in these models. Additionally, we investigated the kinetics of the vascularization process in a colon tumor and a mesothelioma xenograft. In mice bearing a head and neck xenograft, a perfusion study was performed to compare the functionality of the human and mouse tumor vessels.

**Results:**

In patient tumors which successfully engrafted, the human stroma and vessels which were engrafted as part of the original tumor did not survive and were no longer detectable at the time of first passage (15–25 weeks). Uniformly, the stroma and vessels supporting the growth of these tumors were of murine origin. The results of the kinetic studies showed that the loss of the human vessels and vascularization by host vessels occurred more rapidly in a colon tumor (by 3 weeks) than in a mesothelioma (by 9 weeks). Finally, the perfusion studies revealed that while mouse vessels in the periphery of the tumor were perfused, those in the central regions were rarely perfused. No vessels of human origin were detected in this model.

**Conclusions:**

In the tumors we investigated, we found no evidence that the human stromal cells and vessels contained in the original implant either survived or contributed in any substantive way to the growth of these xenografts.

## Background

Pre-clinical investigations of therapeutics for treatment of malignant disease are most often carried out using mouse models. Human tumor xenograft models have traditionally utilized established cell lines, but more recently, engraftment of undisrupted, intact pieces of patient tumors has provided models which more closely resemble the histological organization of patient tumors [1-9]. The maintenance of the architecture of the original patient tumor in combination with the presence of low passage tumor cells results in a model which is highly representative of the diversity of tumors seen in the patient population and, therefore, the benefit of using this model for therapeutic studies is becoming more widely appreciated (e.g. [10]). Recent reports establish two important facts: 1) that primary xenografts genetically resemble the original tumor through several passages and 2) that subcutaneously implanted tumors remain genetically representative of the original [11,12].

In light of the recognized importance of the tumor microenvironment in tumor growth and sensitivity to a variety of treatments, critical issues associated with patient-derived xenografts include determining which components of the original specimen survive and proliferate and which components of the xenografted tumor are derived from the host. Data supporting the presence of host derived components was reported recently by Lin et al. [13]. In this study 43% of the patient pancreatic tumor xenografts analyzed had greater than 50% mouse DNA, indicating that a significant component of the xenograft is derived from the host. In the case of human cell lines, it has been reported that the vessels are of murine origin [14] and the stroma is recruited and derived from the host [15]. However, there are also reports of “mosaic” vessels partially lined by human tumor cells [16] and “vascular mimicry” in which blood cells are seen in channels lined by tumor cells, not endothelial cells [17,18] as well as actual transdifferentiation of tumor cells into cells that are positive for endothelial cell markers in glioblastoma models [19]. In primary tumor xenografts derived from intact pieces of patient tumors, there have been comparatively fewer studies directly addressing the fate of the stromal elements of the original graft or the origin of the stromal elements that support growth of the successful xenografts. A recent review [20] points out that this issue is still controversial and unresolved. In our earlier work [21] describing patient ovarian tumor xenografts, we labeled sections of ovarian tumors for the ALU element of human DNA and demonstrated that although tumor cells were labeled, stromal cells were not, indicating that the stroma of these successful xenografts was murine. Recently, two studies have specifically addressed the question of fate of the human vasculature in the implanted specimens with somewhat different conclusions. Gray et al. [22] studied prostate and renal cell carcinoma (RCC) xenografts. Xenografts were recovered at 30 days and analyzed using antibodies with species-specificity to endothelial cell markers; these authors concluded that in prostate tumors implanted subcutaneously in nude mice, human endothelial cells survive and undergo angiogenesis such that the majority of the proliferating endothelial cells (80%) are of human origin. This was not the case for RCC and these authors conclude there may be differences between tumor types. Sanz et al. [23] performed a similar study on colorectal cancer xenografts and found that in contrast to the results reported for prostate, the human vasculature rapidly disappeared from growing colorectal xenografts so that by day 10, 50% of the vasculature was murine, by day 20 it was predominantly murine and by day 30, no human vessels were detectable. During this time the tumors grew from approximately 4 mm^3^ to 250 mm^3^. More recently, Julien et al. [5] have carried out an extensive characterization of a large cohort of patient colon tumor xenografts and also reported a loss of human stromal cells in early passages of these xenografts as evidenced by *in situ* hybridization with an ALU probe. In agreement with the earlier report, Sanz et al. also found that at 30 days, RCC xenografts contained primarily human vessels, although they did not report the degree of tumor growth achieved during this period. Merk et al. [24], state that in non-small cell lung cancer patient xenografts, the stromal elements are replaced with murine fibroblasts, endothelial and immune cells. Monsma et al. [11] report that the stromal elements persist in xenografts, however, the origin of these elements in engrafted tumors is not specifically addressed. Therefore, it may be that the fate of the human vessels is related to individual tumor types and the timepoint at which the engrafted specimens are examined.

We undertook the current study to directly address the question of the origin of the stromal elements in several different types of xenografted patient tumors with particular attention to the vasculature and identification of the origin of the vessels that support the actual growth of these xenografts. In representative xenografts of eight different tumor types, we found that as the tumors grew to a size to be passaged (approximately 1–1.5 cm diameter), the stroma which developed was not of human origin. Furthermore, in a survey of lung, pancreatic, colorectal and renal cell carcinoma, we found uniformly that the vasculature lacked markers for human endothelial cells and only vessels of murine origin could be identified. Our results support the conclusion that successful engraftment and growth of these patient tumor xenografts depends on recruitment of stroma and new vessels from the murine host. Additionally, we examined the kinetics of vessel recruitment in a colorectal tumor and a mesothelioma and observed that during the initial engraftment, although the time-frame is slightly different, murine vessels gradually became predominant in both tumors. Lastly, we found that in an engrafted head and neck tumor, human vessels were not detected and perfused vessels were of murine origin. Overall, in the tumors we investigated, we found no evidence that the human stromal cells and vessels contained in the original implant either survived or contributed in any substantive way to the growth of these xenografts.

## Methods

### Xenograft model

Fresh surgical specimens of tumors were obtained through the Pathology Resource Network at Roswell Park Cancer Institute through an approved IRB (Institutional Review Board) protocol. The clinical characteristics of the 37 patient tumors whose xenografts were used for various aspects of this study are included in Table 1. Of the 37 patients from whom tumor samples were procured, 9 had received therapy prior to surgery. All specimens were examined by a pathologist and confirmed to contain at least 75% malignant tissue; specimens were transported to our lab and implanted immediately. Tumor specimens were cut into 2×2 mm pieces and surgically implanted subcutaneously in the lower abdomen of 4–6 week old SCID mice as previously described [4,7]. In general, each patient tumor was implanted into 2–3 mice and mice were monitored for tumor growth. Implanted tumors were recovered and passaged into new mice when tumors reached approximately 1–1.5 cm in diameter. Tumors which grew in the second passage were considered successfully engrafted. Pieces of tumors recovered during passaging were formalin fixed, processed for histology and archived. Mice were maintained under sterile conditions in microisolator cages; temperature and light cycles were regulated on a 12 hr. cycle. Mice were fed and had access to water *ad libitum*. All manipulations were carried out aseptically in a laminar flow hood. The RPCI Institutional Animal Care and Use Committee approved all procedures carried out in this study. To monitor development of vasculature during the engraftment process, pieces of a single patient’s tumor were implanted into a group of 12–15 SCID mice. Periodically, mice were removed from this group and tumors were fixed and processed for routine histology. In some cases, tumors were embedded in OCT for cryosectioning.

**Table 1:** Clinical characteristics of tumors and experiments in which corresponding xenografts were used

**Primary site description**	**ID#**	**Exp**	**Sex**	**Age**	**Histology description**	**Grade**	**Path T**	**Path N**	**Path M**	**AJCC stage path**	**Treatment prior to sample**
Esophagus, lower third	12944	2	M	70	Ad, NOS	III	2	1	0	2B	no
Esophagus, lower third	13176	2	M	40	Ad, NOS	III	1	1	1	4	no
Ovary	13575	2	F	67	PSC	III	3C	0	0	3C	Carboplatin/Taxol
Esophagus	13618	2	nd	nd	nd	nd	nd	nd	nd	nd	nd
Ovary	13987	2	F	43	SSPC	III	3C	1	0	3C	no
Pleura, NOS	14967	4	M	70	Meso	nd	3	0	0	3	Radiation and chemotherapy (Alimta & cisplatin)
Pancreas, head of	15010	3	F	74	Inf Duct, NOS	II	3	1	0	2B	no
Pancreas, body of	15406	3	M	74	Inf Duct, NOS	II	2	0	0	1B	no
Kidney, NOS	15773	3	M	69	RCC, NOS	III	3A	0	1	4	no
Kidney, NOS	15818	3	F	57	RCC, Clear cell	III	1B	0	X	1	no
Lung, upper lobe	15946	3	F	73	SCC, NOS	III	2	0	0	1B	no
Pancreas, tail of	16096	3	M	66	Inf Duct, NOS	II	2	0	0	1B	no
Colon, ascending	16115	3	M	59	Ad, NOS	II	X	X	nd	nd	FOLFOX with Avastin
Kidney, NOS	16616	3	M	62	RCC, NOS	III	3B	0	0	3	no
Kidney, NOS	16692	2, 3	M	57	RCC, sarcomatoid	IV	3B	1	X	4	no
Kidney, NOS	16803	3	M	63	RCC, Clear cell	III	1A	X	X	1	no
Colon, ascending	16811	3	F	83	Ad, NOS	II	4	0	0	2B	no
Pancreas, head of	16870	3	F	31	Inf Duct, NOS	III	3	1	0	2B	no
Colon, Sigmoid, NOS	16879	3	F	76	Ad, NOS	II	3	0	0	2A	no
Pancreas, head of	17123	3	M	79	Ad, NOS	III	3	1	0	2B	no
Rectosigmoid junction	17224	2	F	52	Ad, NOS	II	3	1	1	4	no
Lung, lower lobe	17228	3	F	82	CSC	III	2	0	0	1B	no
Colon, ascending	17239	2	M	71	Ad, NOS	II	3	2	0	3C	no
Lung, upper lobe	17246	2, 3	M	53	Ad, NOS	III	3	0	0	2B	no
Lung, lower lobe	17291	1, 3	F	53	Pleomorphic ca	IV	3	0	nd	2B	no
Tonsillar pillar	17307	2	M	67	SCC, NOS	I	0	2B	1	4C	no
Rectosigmoid junction	17346	3	M	57	Ad, NOS	II	3	0	nd	2A	no
Lung, lower lobe	17448	2, 3	M	71	Ad-SCC	II	2	0	X	1B	no
Colon, splenic flexure	17641	1, 2, 3	F	59	Ad, NOS	II	X	X	nd	nd	FOLFIRI & Avastin, Erbitux, irinotecan then oxaliplatin & Xeloda then SAHA, 5-FU and leucovorin.
Cecum	17651	3	F	74	NE	n.d.	4	1	X	4	no
Rectosigmoid junction	18023	4	F	46	Ad, Mucinous	II	X	X	nd	nd	Avastin and FOLFOX then Xeloda and Avastin
Pancreas, overlapping lesion	18254	1, 2	F	69	Inf Duct, NOS	III	3	1	X	2B	no
Floor of mouth, NOS	18316	2	F	47	SCC, krt	I	X	X	nd	nd	Cisplatin and Taxotere
Duodenum	18484	2	M	77	Ad, NOS	II	4	1	0	3	no
Pancreas, head of	18643	2	F	56	Inf Duct, NOS	I	3	1	0	2B	Chemoradiation: total of 5040 cGy with gemcitabine
Pancreas, overlapping lesion	19632	2	F	78	Inf Duct, NOS	III	3	0	nd	2A	no
Palate, soft, NOS	19785	5	M	65	SCC, lg cell, nonkrt	III	0	0	X	nd	Induction chemotherapy

For each tumor listed, the sex, age at first diagnosis, histological description, histological grade, pathological stage and treatment (if any) prior to specimen acquisition is included. The ID# refers to the de-identified number given to the procured samples. Exp indicates the experiments for which the xenograft of each tumor was used: 1- validation of antibody species-specificity, 2-localization of human mitochondria, 3- survey of time to first passage and species identification of vasculature, 4- kinetics of vascularization of engrafted tumors and 5- the perfusion study. For histological descriptions, the following abbreviations are used: NOS- not otherwise specified, Ad- adenocarcinoma, PSC- papillary serous cystadenocarcinoma, SSPC- serous surface papillary carcinoma, Meso- mesothelioma, biphasic, malignant, Inf Duct- infiltrating ductal carcinoma, RCC- renal cell carcinoma, SCC- squamous cell carcinoma, CSC- combined small cell carcinoma, Ad-Sq- adenosquamous, NE- neuroendocrine, krt- keratinizing. For histological grades the following definitions were used: Grade I: well differentiated, Grade II: moderately differentiated, moderately well differentiated, intermediate differentiated, Grade III: poorly differentiated, Grade IV: undifferentiated, anaplastic. Histological staging is per AJCC (American Joint Committee on Cancer), nd- no data.

### Immunohistochemistry

Immunohistochemistry on archival specimens of patient tumors and xenografts derived from those tumors was performed on 5 μ sections of formalin-fixed paraffin embedded tissues (FFPE). Since larger tumors often contained scattered areas of central necrosis, all evaluation of staining was done on selected areas of viable tumor. For performing IHC on FFPE archival specimens, we selected and validated the species-specificity of the following antibodies on FFPE tissue. For identification of vessels of mouse origin, monoclonal rat anti-mouse CD34 was used (AbCam #8158, clone MEC 14.7) with no pretreatment; the secondary antibody was biotinylated goat anti-rat (Pharmingen #554014), and was visualized with either strepavidin (Zymed) or Vector ABC Elite and diaminobenzidine (DAB). For identification of vessels of human origin, polyclonal rabbit anti-human CD31 (Novus Biologicals NB100-2284) was used following antigen retrieval with citrate buffer pH 6.0 (microwave heating 10′ 10% power, followed by a 15′ cooldown); the DAKO Envision kit for Rabbit antibodies was used to visualize bound antibody. Mouse anti-human CD34 (Abcam #ab8536, clone QBEND/10, no antigen retrieval) followed by biotinylated goat anti- mouse secondary (Jackson Immunoresearch #115-065-062) was also used in some experiments. Monoclonal mouse anti-human mitochondria antibody (MAB1273 Chemicon) following antigen retrieval (with EDTA buffer pH 8.0) was used for identification of cells of human origin.

For visualization of perfused vessels, a cohort of mice was engrafted as described above with pieces of a freshly obtained head and neck tumor [9]. Tumor growth was monitored and mice were selected at various times post-engraftment and injected systemically (slowly via tail vein) with 100 μl Dylite 594 conjugated tomato lectin (Lycopersicon Esculentum, Vector). After 2–3 minutes, tumors and intestinal tissue were removed and embedded in OCT. 15-20 μ cryosections were mounted in Vectastain with DAPI. To identify vessels, sections from lectin perfused tissue were counterstained. The following antibodies were used for immunofluorescence: monoclonal mouse anti-human CD34 (Abcam #ab8536, clone QBEND/10) followed by Dylite 488 conjugated Jackson biotinylated goat anti-mouse (Jackson Immunoresearch Cat# 115-485-003) and rat anti-mouse CD31 (Pharmingen Cat# 01951D) followed by Dylite 488 conjugated goat anti-rat (Jackson Immunoresearch Cat# 112-485-167).

### Analysis of blood vessel density

Stained sections were examined under low magnification for representative areas of the tumor. At 20× magnification, five fields were then selected randomly within these areas, vessels were counted and the counts averaged to determine the MVD of either huCD31(+) or msCD34(+) vessels.

## Results

### Stromal elements of successfully engrafted patient tumors do not label for human markers

**Figure 1:**
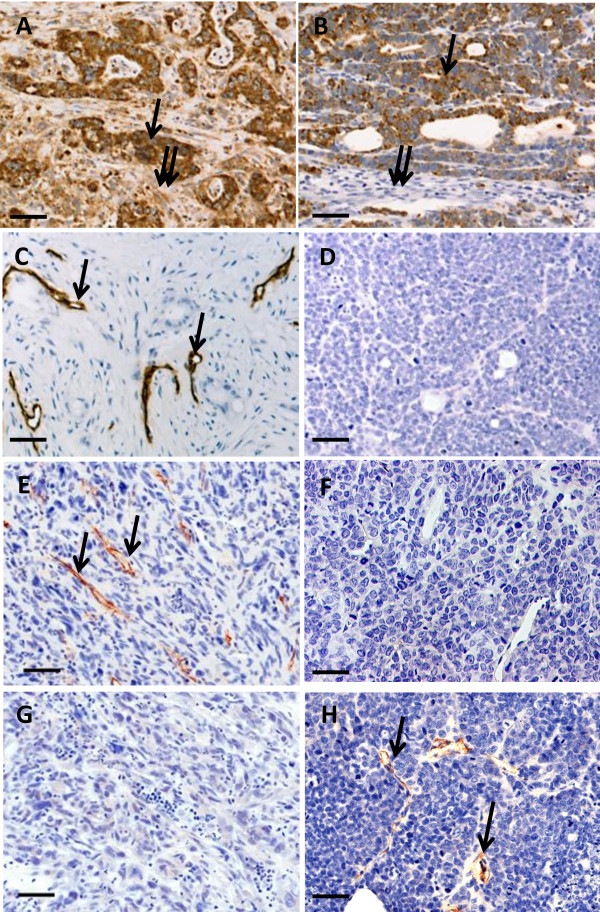
**Antibodies selected for characterization of stromal cells and vasculature in FFPE specimens are species-specific.** (**A**, **B**) Labeling of a patient colon tumor (**A**) and Colo205 human cell line xenograft (**B**) with anti-human mitochondrial antibody (single arrow- tumor cells; double arrows- stroma). (**C**, **D**) Labeling of a patient pancreatic tumor (**C**) and Colo205 human cell line xenograft (**D**) with anti-human CD34. (**E**, **F**) Labeling of a patient lung tumor (**E**) and Colo205 xenograft (**F**) with anti-human CD31. (**G**, **H**) Labeling of a patient lung tumor (**G**) and Colo205 xenograft (**H**) with anti-mouse CD34. (bars = 50 μ).

We were interested in characterizing the process of engraftment and in delineating which components of the microenvironment are derived from the implanted human tumor tissue and which are derived from the murine host. In order to characterize the origin of the diverse stromal cells and the vasculature within several types of patient tumor xenografts using formalin fixed archival tissue, species-specific antibodies were identified and immunohistochemical protocols were developed using FFPE specimens known to contain only human vasculature (patient surgical specimens) or only mouse vasculature (xenograft tumors derived from cell lines). As seen in Figure 1, an antibody specific for a human mitochondrial antigen labeled both tumor cells and stromal cells in a patient specimen of colon carcinoma (Figure 1A) but only the tumor cells in a Colo205 human colon tumor cell line xenograft (Figure 1B). An anti-human CD34 antibody labeled vessels in a specimen of a pancreatic tumor (Figure 1C) but not in a Colo205 xenograft (Figure 1D). Similarly, an anti-human CD31 antibody labeled vessels in a specimen of patient lung tumor (Figure 1E) but not the Colo205 xenograft (Figure 1F). Conversely, an antibody recognizing mouse CD34 did not label the patient lung tumor specimen (Figure 1G), but did label vessels in the Colo205 xenograft (Figure 1H). Therefore, these antibodies were used to identify the species of origin of the vessels in FFPE archival specimens.

**Figure 2:**
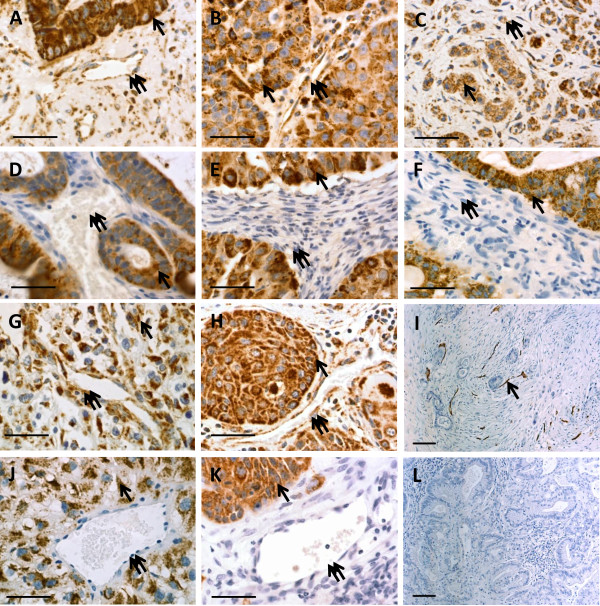
**Stromal elements in patient derived xenografts at termination of the first passage in SCID mice did not label with an anti-human markers; comparison of human markers in patient tumor specimens (A, B, C, G, H, I) and the first passage of the same tumor established as a xenograft (D, E, F, J, K, L).** The anti-human mitochondrial antibody labeled tumor (single arrow) and stromal cells (double arrows) in patient specimens (**A**, **B**, **C**, **G**, **H**), but only tumor cells (single arrow) in the xenografts (**D**, **E**, **F**, **J**, **K**) while the stroma cells (double arrows) were unstained. Similarly, an anti-human CD34 antibody labeled vessels (arrow) in a patient tumor (**I**) but not in the xenograft (**L**). (**A**,**D**- Colon tumor; **B**, **E**- Lung tumor; **C**, F- Pancreatic tumor; **G**, J- RCC; **H**, K- Head & Neck tumor; **I**, L- Pancreatic tumor). (bars = 50 μ; except I, **L** bars = 100 μ).

We carried out an initial characterization of this model in 17 individual patient tumor/ 1^st^ passage xenograft pairs which included 8 different tumor types (3 colon, 2 lung, 3 pancreatic, 1 RCC, 2 head and neck, 1 duodenal, 2 ovarian and 3 esophageal tumors) using blocks of engrafted tumors which had grown to approximately 1–1.5 cm^3^ (a size at which these tumors were reimplanted to a second passage) from our FFPE archive of xenografted tumors. Tumors were often found to have scattered areas of central necrosis and so evaluation of staining patterns was carried out on selected areas of viable tumor. The antibody to the human mitochondrial antigen was used to identify cells of patient (human) origin and revealed that both tumor and stromal cells in the sections of patient specimens were labeled (Figure 2A, B, C, G, H). However, the only labeled cells in tumors which had successfully engrafted in SCID mice were the malignant tumor cells and, notably, stromal elements were not similarly labeled (Figure 2D, E, F, J, K; data for ovarian and esophageal tumors not shown). We also noticed that while endothelial cells of apparent vessels in the patient specimens were labeled by the anti-human mitochondrial antibody, vessels in the xenografts were not. A preliminary analysis of the vascular elements in a patient pancreatic tumor and xenograft with anti-human CD34 antibody confirmed the abundant presence of human vessels in the original surgical specimen, but anti-human CD34 did not label vessels in the xenograft (Figure 2I, L). These observations strongly suggested that the growth of successful xenografts was supported through the recruitment of mouse stromal elements.

### Successfully engrafted patient tumors are characterized by the presence of mouse vasculature, but human vessels are not detectable

It has been suggested that implanted tumors may vary in the degree to which the original human vasculature survives [22,23]. To specifically determine the origin of vessels which support the growth of successfully engrafted patient xenografts, we compared the presence of human and mouse blood vessels in surgical specimens and tumors engrafted in SCID mice as above. We randomly selected surgical specimens and corresponding first passage xenografts of four different types of tumors: colon, pancreatic, lung and renal cell carcinoma (3–7 different tumors in each panel). Serial sections of each surgical specimen and the corresponding xenograft were stained for either huCD31 or msCD34.

Patient xenografts of different tumor types selected for this study grew at slightly different rates and there was variability between tumors with regard to the time to first passage. Colon tumors grew the most rapidly with a mean growth time to passage of 15 weeks. In comparison, the other tumor types grew slightly more slowly. The average time to resection of pancreatic, lung and renal cell carcinomas was 20–25 weeks.

**Figure 3:**
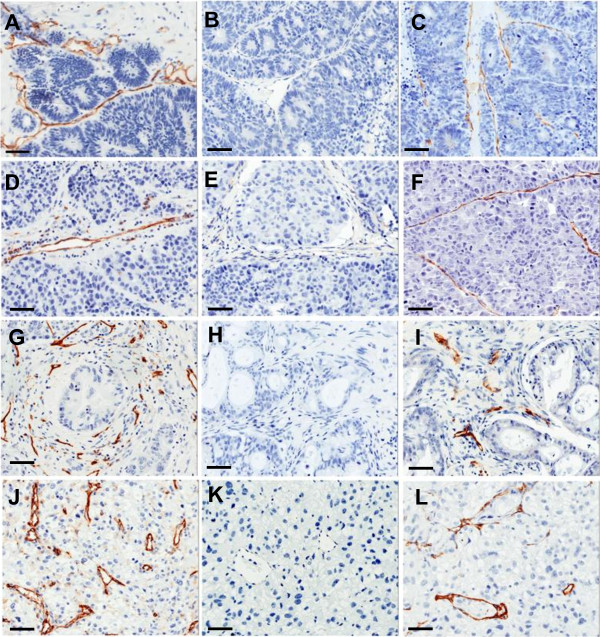
**At the time of second passage, vessels in successfully growing xenografts of four different tumor types surveyed were of murine origin: A-C Colon; D-F Lung; G-I Pancreatic; J-L Renal Cell Carcinoma.** For each tumor type, a representative section of an original patient specimen labeled with anti-huCD31 is shown (**A**, **D**, **G**, **J**). For each tumor, sections of the first passage xenografted tumor when it was resected are also shown; while no huCD31(+) vessels were identified in the xenografts (**B**, **E**, **H**, **K**), msCD34(+) vessels were abundant (**C**, **F**, **I**, **L**). (bars = 50 μ).

**Figure 4:**
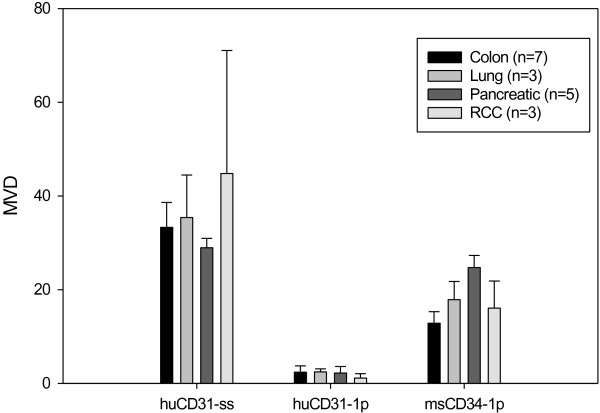
**Successfully engrafted tumors which had grown to a size to be passaged were primarily vascularized by vessels derived from the murine hosts.** Comparison of the microvessel density (MVD) in the patient surgical specimens (ss) and engrafted tumors (-1p) showed that most of the huCD31(+) vessels were lost during tumor growth in the mouse. At the time of first passage, few remaining remnants of huCD31(+) cells were detectable and the major source of blood vessels was the recruitment of msCD34(+) vessels of murine origin. (Values shown are averages of 5 fields/tumor counted at 20×).

In each of the original patient tumor specimens (Figure 3), huCD31(+) blood vessels were apparent (Figure 3A, D, G, J). At the time of resection, huCD31(+) vessels were not detectable (Figure 3B, E, H, K), however msCD34(+) vessels were clearly apparent within the stroma (Figure 3C, F, I, L). Additionally, quantification of microvessel densities in these tumors confirms a preponderance of murine vessels in all successfully engrafted xenografts (Figure 4).

### Kinetics of vascularization of patient tumor xenografts

As discussed above, a survey of tumor types found that by the time successfully engrafted tumors reached a size large enough to be passaged, colon, pancreatic, lung and renal cell tumors were vigorously vascularized by a network of vessels of murine origin. To determine the time-frame of vasculature development in xenografted patient tumors, this process was analyzed using two different patient tumor xenograft models, a mesothelioma and a metastatic colon tumor, for which large amounts of tumor tissue were available enabling the engraftment of many mice with the same primary tumor.

**Figure 5:**
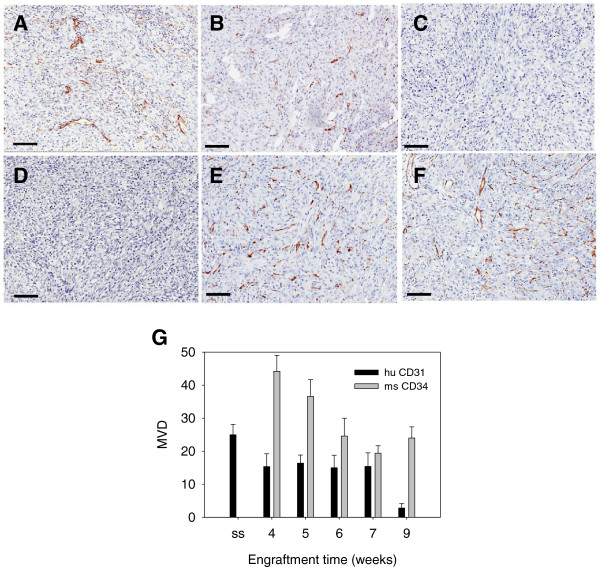
**The growth of a patient mesothelioma xenograft was supported by development of a murine vascular network.** A patient mesothelioma was implanted in a cohort of mice and monitored for tumor growth. Once tumors began to actively grow, representative tumors were resected at weekly intervals and analyzed for vessel content. Staining for huCD31 was prominent in the original patient specimen (**A**), much reduced at 4 weeks (**B**) and negligible at 9 weeks (**C**). In contrast, patient specimens were not stained for msCD34 (**D**), whereas at 4 weeks large numbers of vessels stained for msCD34 (**E**) and by 9 weeks, msCD34 labeled vessels were predominant (**F**). The graph (**G**) summarizes the loss of detectable human vessels and acquisition of murine vessels over a 9 week period. (bars = 100 μ).

A specimen of mesothelioma was engrafted into 12 SCID mice which were then monitored until tumors became palpable at 4 weeks post-implantation. Specimens were then resected and prepared for histology at 4, 5, 6, 7 and 9 weeks post-implantation. This tumor grew rapidly and a first passage tumor was recovered and passaged into new mice on day 45 (7 weeks). This second passage continued to grow and was subsequently resected and re- passaged a third time on day 108. Samples of the original surgical specimen, first passage and second passages were analyzed. The original patient specimen showed widespread labeling of huCD31(+) cells (Figure 5A) and no reactivity with msCD34 (Figure 5D). By 4 weeks, when measureable tumor growth was first detected, there were still detectable huCD31(+) vessels (Figure 5B) while mouse CD34(+) blood vessels were also already abundant in the interior of the xenografts (Figure 5E). At 9 weeks, very few huCD31(+) vessels were detectable (Figure 5C) and the great majority of labeled vessels were msCD34(+) (Figure 5F). Where huCD31(+) blood vessels were seen, they were fragmented and sparsely dispersed (not shown). Blood vessels in established mesothelioma xenografts transplanted into a second passage stained exclusively for msCD34 (data not shown). The number of huCD31(+) or msCD34(+) vessels at each time-point was quantified by counting the number of microvessels in five fields of view at 20× and averaging (Figure 5G). At 4 weeks, the mesothelioma xenograft had a huCD31(+) blood vessel MVD of 15 and a msCD34(+) blood vessel MVD of 44. Over the next five weeks, resected tumors showed diminished numbers of huCD31(+) vessels and the strong presence of msCD34(+) vessels. We observed variability in msCD34(+) MVD from a high at week 5 to an equilibrium at weeks 7–9. This suggests an initial high number of mouse vessels as the original implant is vascularized and then a change in proportion of vessels to tumor cells as proliferation of tumor cells increases.

**Figure 6:**
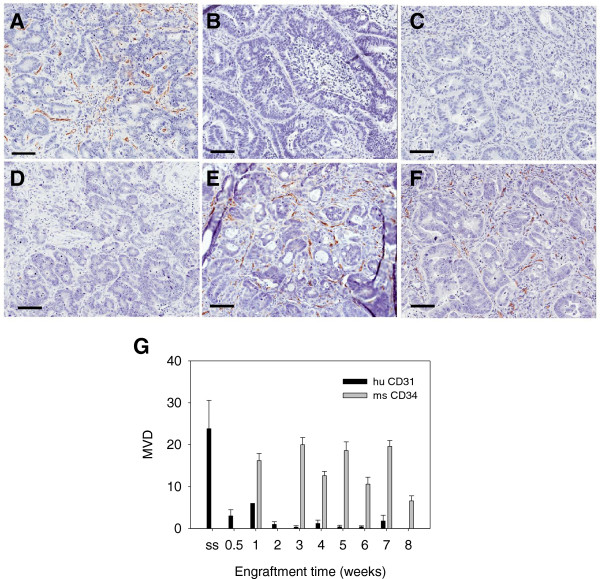
**Vascularization of an engrafted patient colon tumor.** A patient colon tumor was implanted in a cohort of mice and vessel development was analyzed over 8 weeks. Vessels in the original patient specimen labeled strongly for huCD31 (**A**) and not for msCD34 (**D**). Representative sections showing loss of huCD31(+) vessels (**B**- 4 weeks, **C**- 7 weeks) and presence of msCD34(+) vessels (**E**- 4 weeks, **F**- 7 weeks) are shown. The graph (**G**) summarizes this process; huCD31(+) vessels were rapidly lost, and by one week, mouse vessels were the predominate vessels present in the colon tumors (no data for msCD34 at 2 weeks; bars = 100 μ).

To examine an engrafted tumor at earlier timepoints, a specimen of a metastatic colon adenocarcinoma was engrafted into 12 SCID mice and, before apparent tumor growth, colon xenografts were resected at 3 days and one week followed by weekly intervals. As expected, the original patient specimen stained strongly for huCD31 (Figure 6A) and did not stain for msCD34, (Figure 6D). Tumors recovered at 3 days, one week and two weeks were not larger than the original implanted piece, but already showed greatly reduced labeling for huCD31 (see graph Figure 6G). At these early times, although no histological evidence of tumor glandular structures was seen in sections from the implantation sites, msCD34(+) vasculature was seen in the peripheral connective tissue surrounding the implant. By 3 weeks post-implantation, glandular structures were apparent in the periphery of the sample signifying growth of the implanted tumor and these glands were surrounded by vessels staining strongly for msCD34, but huCD31(+) vessels were not detectable. From 4–8 weeks post-implantation, as the tumors grew and the malignant glandular structures expanded, huCD31(+) vessels were not detectable (Figure 6B,C), while an extensive network of msCD34(+) vessels was apparent (Figure 6E,F). In summary, the human vessels were rapidly lost in the early stages of engraftment and vasculature derived from the mouse predominated during the growth phase (Figure 6G).

### Functional vasculature during growth of xenografts is of mouse origin

**Figure 7:**
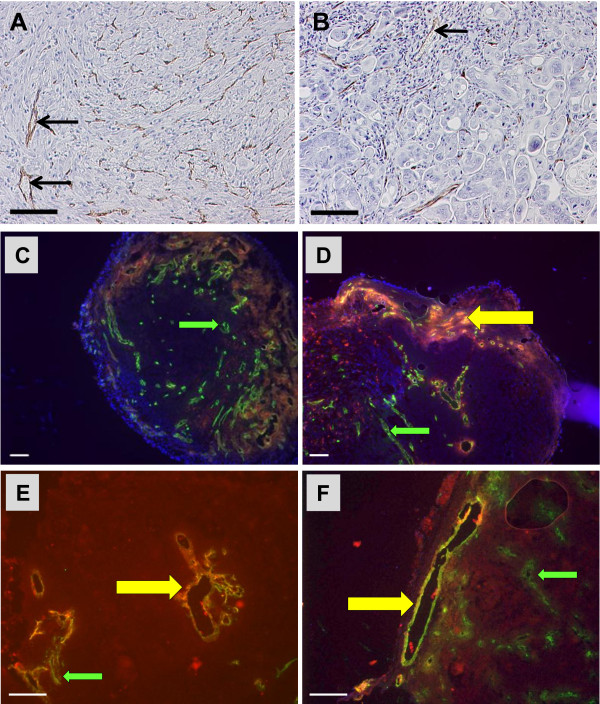
**Murine vessels were functional in xenografted tumors.** (**A**, **B**) Paraffin sections of first passage xenografts of the mesothelioma (**A**) and colon (**B**) tumors labeled for msCD34 show that murine vessels often contained red blood cells (arrows). (**C**-**F**) A patient head and neck tumor was engrafted into a cohort of mice and the growth of xenografts was monitored beginning 19 days after implantation when tumors became palpable. Functional analysis of vessels was carried out on tumors of different sizes. Animals were injected with Dylight-594 labeled tomato lectin (red) and frozen sections were prepared. Representative sections stained for msCD31 visualized with a Dylite 488 (green) secondary antibody are shown: volumes were (**C**) 19 mm^3^, (**D**) 75 mm^3^, (**E**) 190 mm^3^ and (**F**) 600 mm^3^. In each case, msCD31(+) vessels (green arrows) were seen in the tumors, both centrally and in the periphery. Perfused vessels which were msCD31(+) were seen in the periphery of tumors (**D**-**F**; yellow arrows). Non-perfused vessels were also observed in the central portions of these tumors. (bars = 100 μ).

In the slides of the mesothelioma and colon xenografts described above, vessels of murine origin were often seen to contain red blood cells (RBCs) supporting the idea that these murine vessels were functional and contiguous with adjacent host blood vessels (Figure 7A, B). To further examine the extent to which human vs. mouse vessels were functional in growing xenografts, we engrafted a cohort of mice with a head and neck tumor and tumor growth was monitored. At intervals between 4 weeks to 8 weeks post-implantation, tumors were measured and mice were injected systemically (by tail vein) with Dylight 594 labeled tomato lectin (red fluorescence) to visualize perfused vessels; tumors and intestinal tissue (as a control for successful perfusion) were resected and embedded in OCT. For this study, tumors were selected for analysis based on growth (as indicated by volume) rather than time after implantation. Analysis of tumors was initiated once vigorous growth began; these tumors ranged in size from 32–75 mm^3^ on day 20 after implantation when palpable tumors became apparent, to 200–800 mm^3^ after day 42. Freshly obtained cryosections were prepared and perfused vessels were visualized by fluorescence microscopy. Immunofluorescence staining was performed on frozen sections using either anti-msCD31 or anti-huCD34 to determine the origin of perfused vessels. We observed that, as tumors grew, perfused vessels were clearly evident in the connective tissue surrounding the tumor (Figure 7C-F). At all stages of growth, these perfused vessels adjacent to the tumor stained strongly for msCD31. Vessels positive for msCD31were also visualized within the tumors (Figure 7C-D, green arrows), while no vessels in these tumors labeled for huCD34 (data not shown). Lectin perfused vessels were sparse within tumors of all sizes, but when detected, these were observed to co-stain for msCD31 (Figure 7D-F, yellow arrows). These results suggest that even when abundant vessels are recruited, as the tumor grows, well perfused vessels are mainly limited to the periphery.

Overall, these results support the conclusion that growth of patient xenografts in SCID mice is supported by vasculature recruited from the murine host. Although fragments of the vessels originally present in the human tumor implant could sporadically be visualized, we found no evidence that these residual fragments were functional or sustained the actual growth phase of the xenografts.

## Discussion

Although there is general acceptance of the fact that patient derived tumor xenografts provide a model in which the histology and genetics of the original tumor is maintained, there is not yet a clear understanding of the actual process of engraftment and the fate of various components of the patient tumors. For example, a recent review discussing the benefits of the patient tumor xenograft model for predicting drug responses states that while cell line xenografts lack human stroma and immune cells, in patient xenografts the tumor and stromal components are from the same individual [25] although these authors also state that this may vary depending on the strain of mouse used. In a recent review, Tentler [20] points out that the answer to the question of how long the human vasculature and other components survive in patient xenografts is controversial. Overall, in light of the recognized importance of the tumor microenvironment in tumor physiology and response to therapeutics, it is critical to better understand this process. Additionally, as patient xenografts are increasingly used to study therapeutic efficacy, this could be particularly important in therapeutic experiments in which the vasculature or other stromal elements are being targeted.

For this study, we used representative first passage xenografts of eight different tumor types. Our findings presented here support the conclusion that, in patient xenografts, the malignant cells in the patient tumor survive while components of the original stroma do not. In identifying the origin of the vasculature in a survey of four different tumor types (pancreatic, lung, colon and renal cell carcinoma) we found that the only vessels detectable at the time of first passage were host-derived. Our kinetic studies of two different tumors show that although the time frame of host vessel recruitment may differ slightly between tumor types, as previously reported for RCC and colon tumors [22,23], ultimately the human vessels were lost and tumor growth was supported by host vessels. Also as seen previously, some of these vessels contain RBCs attesting to their functionality, and we have confirmed this by dye perfusion experiments. Thus the general consensus of the few reports in which this question has been studied specifically in patient tumor xenografts is that under experimental protocols in which undisrupted pieces of patient tumors are directly engrafted into immunodeficient mice, the engraftment and growth of the xenografts is largely supported by recruitment and incorporation of host stromal elements, particularly the vasculature.

There are cases where investigators would like to be able to establish xenograft models with human stromal elements and this has been attempted in different ways. One approach has been to create chimeric mice by first engrafting pieces of human skin onto immunodeficient mice. Using this approach, melanoma cell lines implanted in human skin grafts survived for several months and human vessels were observed to anastomose with host vessels and support tumor growth [26]. Similarly, Tahtis et al. [27] successfully engrafted human skin onto SCID mice and subsequently implanted MCF7 cells in the xenografted skin. The tumors grew and developed extensive stroma and vasculature of human origin which remained viable as murine vessels grew into the tumor, and both were identifiable at 10 weeks after engraftment, although eventually murine vessels formed the majority of the vasculature. Early attempts to engraft human skin into nude mice also demonstrated that the human vessels were gradually replaced by murine vessels [28]. This type of SCID mouse/human skin model has been used to study anti-angiogenic treatments in xenografted breast cancer cell line models [29,30]. Human bone containing human blood vessels has also been xenografted in SCID mice and been shown to support tumor formation by human prostate cancer cells, which do not similarly colonize mouse bone [31].

Recently, a model of human angiogenesis within intact pieces of prostate tumor has been developed to facilitate study of anti-angiogenic therapy for prostate cancer [22,32]. Prostate endothelial cells express androgen receptor and this model depends on the implantation of slow release testosterone pellets prior to engraftment. These authors demonstrate a burst of angiogenesis of the endogenous human vessels during the first 14 days after implantation that was significantly less in mice without the testosterone pellets; this activity is prostate specific and was not observed in RCC xenografts. Between 15–30 days, this new vasculature matured, becoming associated with pericytes and showed reduced leakage. These authors point out, though, that this angiogenic burst is only observed following the initial engraftment and this model is not amenable to serial passaging, so that this model provides a window of opportunity for observing stromal/tumor cell interactions and performing pre-clinical therapeutic testing on patient derived prostate tumors.

## Conclusions

In summary, although there are some special situations in which human tumor xenografts may be supplied, at least for a while, with human derived vessels, we observed overall that in the patient tumor xenograft model in which intact tumor pieces are directly engrafted into naive, unmanipulated SCID mice, vessels of mouse origin can be detected in the periphery of the tumor at very early stages of engraftment. These can be seen even while residual human vessels are still detectable. However, this situation rapidly changes as the number of viable murine vessels increases and the human vessels become fragmented. We found no evidence to suggest that human vessels contribute significantly to the engraftment process in the models we examined. Furthermore, we have never observed any human vessels in subsequently passaged tumors. Because persistence of human vessels seems to be an exception rather than the rule, the characterization and timeframe of stromal and vascular development should be validated for each individual experimental tumor model. This is particularly important when using these models for testing therapies directed at stromal components and when such models are being used for genetic analyses.

## Abbreviations

FFPE: Formalin-fixed paraffin embedded; IHC: Immunohistochemistry microvessel density; OCT: Optimal cutting temperature embedding compound; RBC: Red blood cell; RCC: Renal cell carcinoma; SCID: Severe combined immunodeficiency.

## Competing interests

The authors declare that they have no competing interests.

## Authors’ contributions

BLH established xenografts, designed and directed the study and analysis of results, developed immunohistochemical protocols and drafted the manuscript. NP contributed to design of the study, carried out immunohistochemistry and participated in drafting of the manuscript. HT participated in establishing and maintaining xenografts, collecting specimens and carrying out perfusion assays. JH carried out the perfusion experiment. MV contributed to development of immunohistochemistry protocols. WB assisted in data collection and evaluation of patient tumor characteristics. RP established xenografts and collected specimens. EAR conceived of this study, participated in its design and coordination and helped to draft the manuscript. All authors approved the final manuscript.
